# Characterization and Expression of Genes Involved in the Ethylene Biosynthesis and Signal Transduction during Ripening of Mulberry Fruit

**DOI:** 10.1371/journal.pone.0122081

**Published:** 2015-03-30

**Authors:** Changying Liu, Aichun Zhao, Panpan Zhu, Jun Li, Leng Han, Xiling Wang, Wei Fan, Ruihua Lü, Chuanhong Wang, Zhengang Li, Cheng Lu, Maode Yu

**Affiliations:** 1 State Key Laboratory of Silkworm Genome Biology / College of Biotechnology, Southwest University, Chongqing, China; 2 Citrus Research Institute, Chinese Academy of Agriculture Sciences, Chongqing, China; 3 Institution of Sericulture and Apiculture, Yunnan Academy of Agricultural Sciences, Yunnan, China; Zhejiang University, CHINA

## Abstract

Although ethylene is well known as an essential regulator of fruit development, little work has examined the role ethylene plays in the development and maturation of mulberry (*Morus* L.) fruit. To study the mechanism of ethylene action during fruit development in this species, we measured the ethylene production, fruit firmness, and soluble solids content (SSC) during fruit development and harvest. By comparing the results with those from other climacteric fruit, we concluded that *Morus* fruit are probably climacteric. Genes associated with the ethylene signal transduction pathway of *Morus* were characterized from *M*. *notabilis* Genome Database, including four ethylene receptor genes, a EIN2-like gene, a CTR1-like gene, four EIN3-like genes, and a RTE1-like gene. The expression patterns of these genes were analyzed in the fruit of *M*. *atropurpurea cv*. *Jialing* No.40. During fruit development, transcript levels of *MaETR2*, *MaERS*, *MaEIN4*, *MaRTE*, and *MaCTR1* were lower at the early stages and higher after 26 days after full bloom (DAF), while *MaETR1*, *MaEIL1*, *MaEIL2*, and *MaEIL3* remained constant. In ripening fruit, the transcripts of *MaACO1* and *MaACS3* increased, while *MaACS1* and *MaACO2* decreased after harvest. The transcripts of *MaACO1*, *MaACO2*, and *MaACS3* were inhibited by ethylene, and 1-MCP (1–methylcyclopropene) upregulated *MaACS3*. The transcripts of the *MaETR*-like genes, *MaRTE*, and *MaCTR1* were inhibited by ethylene and 1-MCP, suggesting that ethylene may accelerate the decline of *MaETRs* transcripts. No significant changes in the expression of *MaEIN2*, *MaEIL1*, and *MaEIL3* were observed during ripening or in response to ethylene, while the expressions of *MaEIL2* and *MaEIL4* increased rapidly after 24 h after harvest (HAH) and were upregulated by ethylene. The present study provides insights into ethylene biosynthesis and signal transduction in *Morus* plants and lays a foundation for the further understanding of the mechanisms underlying *Morus* fruit development and ripening.

## Introduction

In climacteric fruit, ethylene is necessary for the initiation of fruit ripening and senescence because it drives the majority of the ripening processes, such as fruit softening and cell wall disassembly [1–2]. The effects of ethylene in fruit mainly depend on its biosynthesis and signal transduction during fruit development.

The synthesis of ethylene begins with the production of S-adenosylmethionine (SAM) which is catalyzed by S-adenosylmethionine synthetase (SAM synthetase) from methionine. SAM is then metabolized to 5-methylthioadenine (MTA), which is incorporated into the methionine cycle to recover the sulfur atom and 1-aminocyclopropane-1-carboxylic acid (ACC); this reaction is catalyzed by ACC synthase (ACS). Finally, in the presence of oxygen, ACC is oxidized by ACC oxidase (ACO) to yield ethylene and CO_2_ [3].

ACS and ACO are the key enzymes in ethylene production and are encoded by the *ACS* and *ACO* gene families, respectively. The expression profiles and regulation mechanisms of *ACS* and *ACO* genes in fruit have been investigated in plants. In tomato (*Solanum lycopersicum* L.), nine *ACS* genes and five *ACO* genes have been characterized and shown to be differentially expressed during fruit ripening [4]. During development (System I), lower and auto–inhibitory levels of ethylene are synthesized by *LeACS1A* and *6* and in conjunction with *LeACO1*, *3*, and *4*. At the transition stage, *LeACS4* is induced and a large increase in auto-catalytic ethylene is initiated. *LeACS2* and *4*, in conjunction with *LeACO1* and *4* are then responsible for the high ethylene production throughout System II [4]. *ACS* and *ACO* genes have previously been used as targets to suppress the production of ethylene and delay the postharvest ripening and senescence of fruit [5–7].

The signal transduction of ethylene is well understood in higher plants. Ethylene signals are transported via five elements: ethylene receptor (ETR), constitutive triple response 1 (CTR1), ethylene insensitive 2 (EIN2), ethylene insensitive 3/EIN3-like protein (EIN3/EILs), and ethylene response factor (ERF). Ethylene is perceived by a family of endoplasmic reticulum (ER)–localized receptors (ETR) that are similar in sequence and structure to bacterial two-component histidine kinases [8]. These ETR then activate CTR1, a Raf-like serine-threonine kinase that acts as a negative regulator of ethylene responses, which directly interacts with the ETR and transmit the signal to EIN2 [9]. EIN2 is a central regulator of the signaling pathway located in the ER. In the presence of ethylene, CTR1 is suppressed, and leading to the dephosphorylation of EIN2 and the slicing of its C-terminal end (CEND), which enters into the nucleus, and activates EIN3/EILs. EIN3/EILs eventually stimulate the expression of ERF and other responsive genes [10–11].

These elements of ethylene signaling have been isolated from several fruit plants to date, and their expression and function have been investigated. The transcript levels of ETR are correlated with the accumulation of ethylene during fruit development, while the concentration of ETR protein decreases gradually [12–13]. ETR protein probably degrades rapidly in the presence of ethylene and this changeable concentration may modulate the onset of fruit ripening by measuring cumulative ethylene exposure, as the expression of *ETR* transcripts causes the production of new ETR protein [13–14]. The expression of *CTR1* tends to increase during fruit maturation in kiwifruit, pear, and plums and responds to ethylene and 1–methylcyclopropene (1-MCP), but expression of this gene is steady in peach and apple [15–18]. EIN2 is also closely related to the regulation of the fruit ripening process, the silencing of *EIN2* will delays fruit development and ripening and greatly reduces the expression levels of ethylene–related and ripening–related genes compared to the control fruits [19–20]. EIN3/EILs are expressed differentially during the development of fruit, and the reduction in tomato *LeEIL* gene expression using antisense technology causes ripening–impaired phenotypes [21–22]. EIN3/EILs family proteins regulate the expression of responsive genes in fruit, including *ERF*, *ACO*, xyloglucan endotransglycosylase gene (*XET*), and cell wall–modifying genes [23–24].

Mulberry (*Morus* L.) provides the optimal food (leaves) for the domesticated silkworm (*Bombyx mori* L.). *Morus* also has multiple uses in ecology, pharmaceuticals, and traditional Chinese medicine, and *Morus* fruit is very popular due to its unique flavor [25]. However, ethylene biosynthesis and signaling perception in *Morus* remain relatively unexplored. In 2008, Pan and Lou [26] became the first to characterize an *ACO* gene from *Morus*, which may be associated with tissue aging or senescence and stress responses. In our previous study, five *MaACS* and two *MaACO* genes were screened from the *M*. *notabilis* Genome Database, and their expression profiles were determined to be related to fruit development, and response to abscisic acid and ethephon [27]. These studies were insufficient to clarify the functions of ethylene during development of *Morus* fruit. In this study, 11 putative genes associated with the ethylene signal transduction pathway were identified in the *M*. *notabilis* genome. The expression patterns of these genes during *M*. *atropurpurea cv*. *Jialing* No.40 fruit development were studied using real-time PCR. These expression patterns were also examined in fruit treated with ethylene and the receptor inhibitor 1–MCP.

## Materials and Methods

### Screening and identification of the genes involved in signal transduction

The protein sequences of the genes involved in the signal transduction from other plant species were downloaded from GenBank (http://www.ncbi.nlm.nih.gov/Genbank/), and were used as queries to blast against *M*. *notabilis* Genome Database (http://morus.swu.edu.cn/morusdb/). This yielded non-redundant full mRNA sequences. The candidate genes were identified using BLASTN and SMART (http://smart.embl-heidelberg.de/).

The amino acid sequences involved in the ethylene signaling pathway were downloaded from NCBI and used for alignment with putative protein sequences of *Morus* signal transduction elements given by MUSCLE 3.6, and the alignment was corrected manually using BioEdit software. And a phylogenetic tree was constructed by MEGA 5.0.

### Plant material and treatments

The mulberry tree *M*. *notabilis*, which is used for genome sequencing, is an isolated wild mulberry species with a chromosome number of 14 [25, 28]. The fruits of *M*. *atropurpurea* cv. *Jialing* No.40, a new cultivated variety for obtaining mulberry fruits, were used for fruit evaluation and quantitative reverse transcriptase polymerase chain reaction (qRT-PCR) in this study. Three different batches of *M*. *atropurpurea* cv. *Jialing* No.40 fruit were collected in the *Morus* garden at Southwest University, Chongqing, China. The batch 1 fruits were collected at 2, 8, 14, 21, 26, 31, 34 and 37 days after full-bloom (DAF), with the last sampling date set at full maturity, and were used for genes cloning and qRT-PCR. The batch 2 fruits were collected at 34 DAF, the fruit were at early ripening stage for this batch, and were incubated in a container with C_2_H_4_ (20 ppm) and 1-MCP (0.5 μl L^−1^ SmartFresh^TM^) at 23°C for 12 h. Then the containers were opened to release the remaining C_2_H_4_ and 1-MCP. Finally, the treated fruits were incubated continually at 23°C for 0, 12, 24, and 36 h. The batch 3 fruits were collected at 21, 26, 31, 34 and 37 DAF, with the last sampling date set at full maturity, and their respiration rates were measured.

### Measurements of ethylene production, fruit firmness, soluble solids content (SSC) and respiration rate

Eight headspace bottles (20 ml), each containing one fruit, were used to evaluate the fruit ethylene production rate. The sealed bottles were held at 23°C for 2 h, and then 1 ml of headspace gas was removed using an airtight syringe. The ethylene was measured using a gas chromatograph (GC2010; Shimadzu, Japan) fitted with a flame ionization detector. The temperatures of the oven, injector and detector were 110°C, 130°C, and 200°C, respectively, and nitrogen was used as the carrier gas at 40 ml min^−1^.

Fruit firmness was measured using a CT3 texture analyzer (Brookfield, USA) fitted with a 2 mm diameter probe. The probe was pressed into eight different fruits to a depth of 3 mm in a single smooth motion. Juice samples of 200 μl pressed from *Morus* fruit were used to measure SSC by using a PAL-1 refractometer (Atago, Japan).

Respiration rate was measured as CO_2_ production. Three replicates of *Morus* fruit were weighed before being sealed in a 5 L container at 25°C. Increased CO_2_ concentration in the container was monitored using a CO_2_ monitoring instrument (RD-7AG; Nanjing Analytical Instrument Factory Co., Ltd. China). The results were calculated as mg CO_2_ kg FW^−1^ h^−1^.

### Isolation of RNA, and synthesis of the first strands of cDNA

Total RNA of fruit was extracted using the RNA Extraction Kit *Transzol* Plant (TransGen Biotech, China). DNase I (Takara, Japan) was used for removing contaminating DNA. The cDNA was synthesized from DNA-free RNA with the Reverse Transcriptional M-MLV (Promega, USA). Ten-fold diluted cDNA was used in qRT-PCR.

### Quantitative RT-PCR expression analysis

The primers (listed in Table 1) were used to isolate the genes involved in ethylene signal transduction in *M*. *atropurpurea* cv. *Jialing* No.40. The purified PCR products were inserted into the pMD19-T simple vector (Takara, Japan) and the sequences were confirmed by sequencing.

**Table 1:** Primers used for cloning the genes involved in the ethylene signal transduction in *Morus atropurpurea* cv. *Jialing* No.40.

**Genes**	**Primers (forward/ reverse)**
*MaETR1*	5’-TGAGTGTTAAGACCAGGGAGCT-3 5’-GAAATCATTGCGAGCACGAATA-3
*MaETR2*	5’-GCTTCTGGAGCATCGAGAACAT-3’ 5’-CAACCCTGTTTATGTCATCATC-3’
*MaERS*	5’-GGCATGTTAGAATGCTGACTCA-3’ 5’-AGCAAGGAAATCATTCCGAGCG-3’
*MaEIN4*	5’-CGGCAATTCGGATGCCAATGCT-3’ 5’-ACCACTCTCTGAGAAAACCCGA-3’
*MaCTR1*	5’-ATGGAAATGCCCGGTAGGAGAT-3’ 5’-TCAGCTTAGTAATTGCATATTTGGATGG-3’
*MaEIL1*	5’-ATGGGCATTTTCGAAGAGCT-3’ 5’-TCAGATGTACCAGAGGGAGA-3’
*MaEIL2*	5’-ATGGGGTTTTCTGGTAAT-3’ 5’-TCAGAGGTACCAGAGCGAGA-3’
*MaEIL3*	5’-ATGGCCTATGACTCATTGGT-3’ 5’-TTATGCTCCGAAGTAATGAATC-3’
*MaEIL4*	5’-ATGGTGAAGTTCCATGAAGA-3’ 5’-TCACTCATATCTCGAATCCC-3’
*MaRTE*	5’-ATGGATTCTGATGCAGACACTG-3’ 5’- CTACAACTGAATTAAATTCTTG-3’

The primers (listed in Table 2) used for qRT-PCR were designed based on the gene sequences obtained from *M*. *atropurpurea* cv. *Jialing* No.40. The qPCR mixture (20 μl total reaction volume) composed of 10μl of 2× SYBR^®^ Premix Ex Taq II ^®^ (Takara, Japan), 0.8 μl of each primer (10 μM), 0.4 μl of 50× ROX Reference Dye, 2 μl of cDNA, and 6 μl of ddH_2_O. The thermal parameters for qRT-PCR were: 95°C for 30 s, followed by 40 cycles of 94°C for 5 s and 60°C for 30 s, with a final extension at 72°C for 10 min. *MaACTIN3* (HQ163775.1) gene was used as an internal control. All data was analyzed using 2^−ΔCt^ method.

**Table 2:** Primers used for qRT-PCR in *Morus atropurpurea* cv. *Jialing* No.40.

**Genes**	**Primers (forward/ reverse)**
*MaACO1*	5’-AAGGTGATGAGGGAATTTGC-3’ 5’-GGTCCCTTTGAGCCATAGAA-3’
*MaACO2*	5’-TCTTGGACTGGAGAAAGGGT-3 5’-CCCTTGATCAGGTCTGGTTT-3
*MaACS1*	5’-GCAACACTGACCTCATCCAC-3 5’-TGCGAGGATACTAATCCGAA-3
*MaACS3*	5’-TGGCTTCCAAATCACTGAAG-3 5’-GGTTGTGTTCGTTCGATGTC-3
*MaETR1*	5’-GCCAGACTACGGCCTCTTAG-3 5’-TGGCCAGTCATTTATTTGGA-3
*MaETR2*	5’-GCAGTACTGTTCGGTTTGGA-3’ 5’-CCTCACAACATCAGGGTCAG-3’
*MaERS*	5’-AAGTTGGATCCTCCATTCCA-3’ 5’-GTGGCACATATCTCCCAACA-3’
*MaEIN4*	5’-TTTCTCATGCCGATGTTCTT-3’ 5’-CCATCATCGCATTCTTCTTG-3’
*MaEIN2*	5’-TGATTTCCATGGGCAACTAA-3’ 5’-AATTCCTTTGCGGTGGTATC-3’
*MaCTR1*	5’-GCAGACGGAGGAGAGTTACC-3’ 5’-CGTCCTTATGGAGGATTCGT-3’
*MaEIL1*	5’-GGAGGCTTTAGAGCATGAGC-3’ 5’-TCAATAGCATGCGGTCTCTC-3’
*MaEIL2*	5’-AACGATGATGCTGGATCAGA-3’ 5’-ACGGTATGGGCAATTCATTT-3’
*MaEIL3*	5’-AACGGGACATCTGGGATAAC-3’ 5’-ATCAGTGCCATCAACGTCAT-3’
*MaEIL4*	5’-AGTCATTGTGTTTGCAAGGC-3’ 5’-TTGGTGAGTTGGGAAAGGAT-3’
*MaRTE*	5’-ATTTCGTGTGCGTTGACAAT-3’ 5’-TCTCTCTTTCGGGTTGATCC-3’

## Results

### Identification of genes involved in ethylene signaling in the *M*. *notabilis* genome

Five *MaACS* and two *MaACO* genes involved in ethylene biosynthesis in *Morus* have been reported by our previous research [27]. In this study, we identified 11 putative ethylene signaling genes in *M*. *notabilis* using bioinformatic methods. These were four ethylene receptor genes (*MnETR1*, *MnETR2*, *MnERS*, and *MnEIN4*), an EIN2-like gene, a CTR1-like gene, four EIN3-like genes (*MnEIL1*, *MnEIL2*, *MnEIL3*, and *MnEIL4*), and a Reversion-to-ethylene sensitivity 1-like gene (*MnRTE*) (Table 3; Table 4).

**Table 3:** Genes involved in ethylene signaling in *Morus notabilis* genome.

**Gene family**	**Number**	**Gene Names**	**Accession No.** ^a^	**Scaffold(strand): start-end**
Ethylene receptor	4	*MnETR1*	Morus018344	scaffold521(-):266232–271313
		*MnETR2*	Morus008145	scaffold606(-):247113–250061
		*MnERS*	Morus007485	scaffold1526(+):209537–212408
		*MnEIN4*	Morus024538	scaffold205(-):594177–596734
Ethylene insensitive 2	1	*MnEIN2*	Morus024376	scaffold342(-):402131–407744
Constitutive triple response 1	1	*MnCTR1*	Morus003569	scaffold548(+):106459–113027
Ethylene insensitive 3	4	*MnEIL1*	Morus002490	scaffold3287(-):31544–33397
		*MnEIL2*	Morus002491	scaffold3287(-):50362–52185
		*MnEIL3*	Morus007978	scaffold18(-):151180–153015
		*MnEIL4*	Morus016593	scaffold841(+):86287–87918
Reversion-to-ethylene sensitivity 1	1	*MnRTE*	Morus026815	scaffold184(+):1246802–1247817

^a^Accession No. are from http://Morus.swu.edu.cn/Morusdb/

**Table 4:** Information of genes involved in ethylene signaling in *Morus notabilis*.

**Gene Names**	**Genome length**	**CDS length** ^a^	**Exon Number** ^**b**^	**Proteins Size**	**MW (Da)**	**PI**
*MnETR1*	5082	2340	7	779	87392.90	7.93
*MnETR2*	2949	2382	2	793	88251.87	6.62
*MnERS*	2872	1853	5	617	68930.31	5.84
*MnEIN4*	2558	2295	2	764	86210.43	6.95
*MnEIN2*	5614	3921	7	1306	142614.61	5.72
*MnCTR1*	6569	2592	17	863	93948.76	6.26
*MnEIL1*	1854	1854	1	617	69703.57	5.74
*MnEIL2*	1824	1824	1	607	68945.70	5.46
*MnEIL3*	1836	1836	1	611	68635.59	5.66
*MnEIL4*	1632	1632	1	643	62078.05	4.74
*MnRTE*	1016	693	2	230	25964.05	6.64

^a^ The CDS length was predicted according to the predicted *Morus* genes.

^b^ The number of exon was predicted based on the *Morus* genomic data.

### Structure and phylogenetic analysis

**Fig 1:**
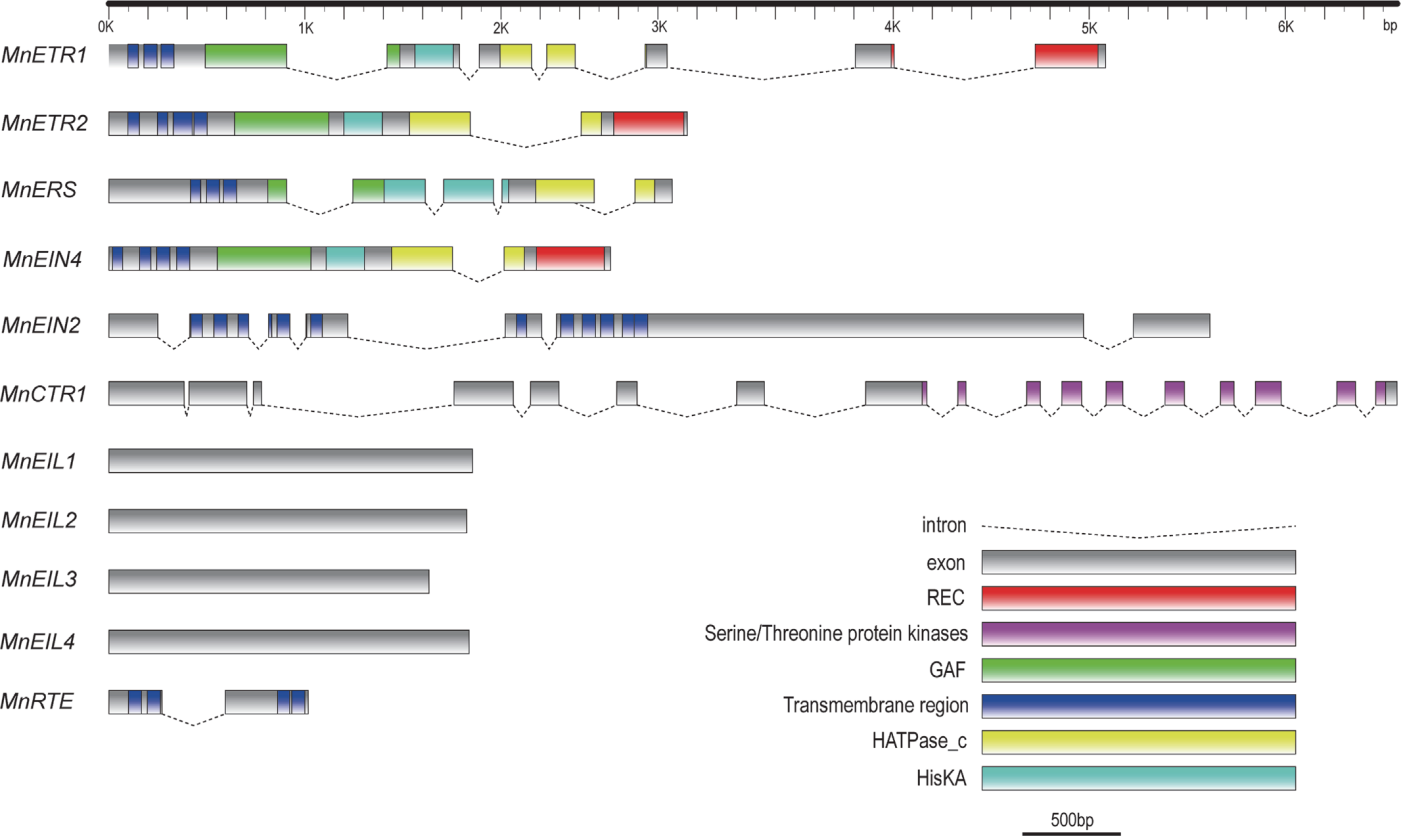
Gene structure and predicted functional domains of *Morus notabilis* ethylene signaling genes. Gene structures were obtained by aligning the cloned cDNA sequences with the *M*. *notabilis* genome data and functional domains were predicted in SMART (http://smart.embl-heidelberg.de/smart/set_mode.cgi?NORMAL=1). Gene structures were displayed by Fancy Gene (http://bio.ieo.eu/fancygene/)

**Fig 2:**
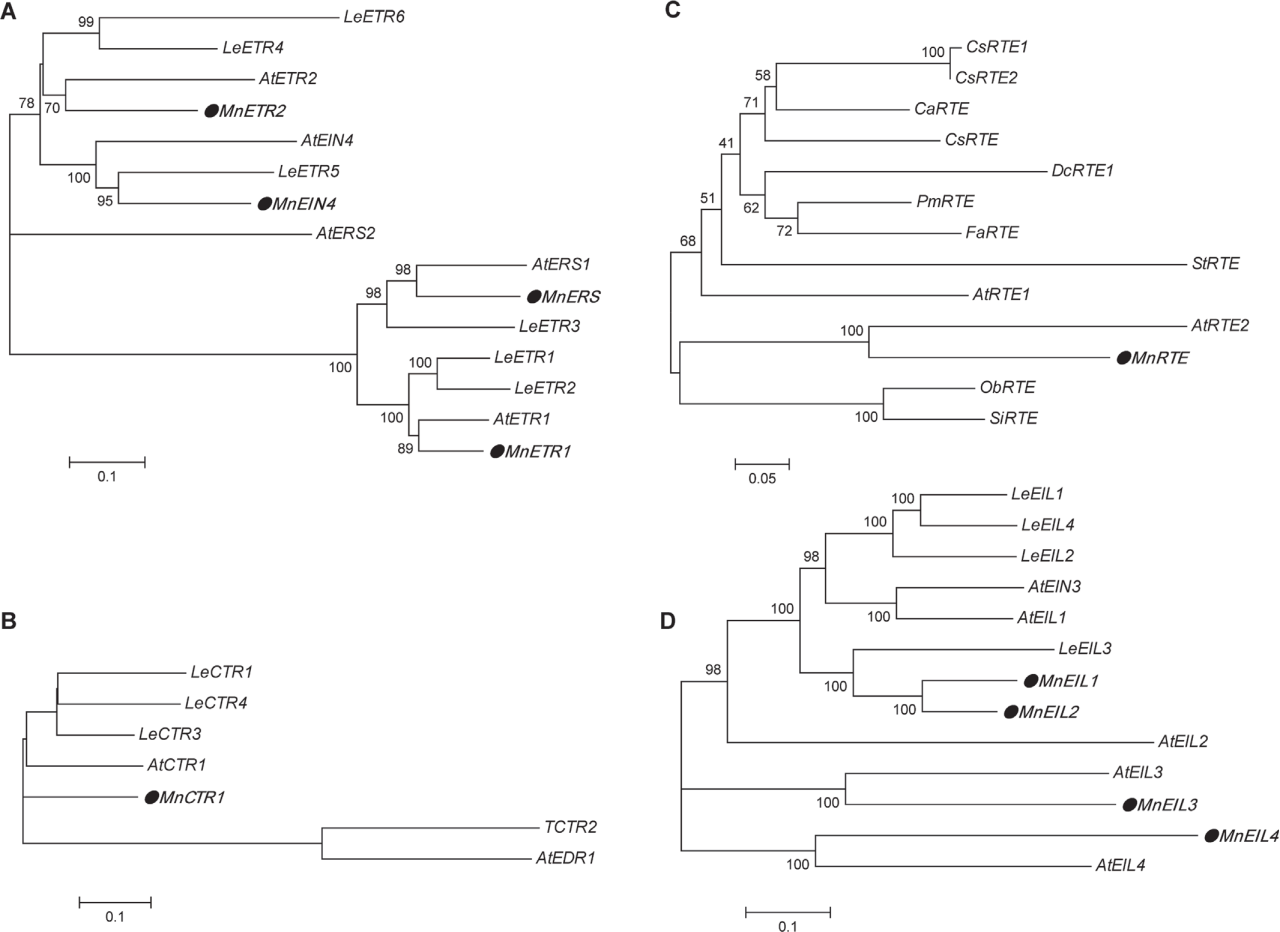
Phylogenetic analysis of MnETRs (A), MnCTR1 (B), MnRTE (C), and MnEILs(D). The amino acid sequences were analyzed with MUSCLE 3.6 and the phylogenetic tree constructed with MEGA 5.0 using a bootstrap test of phylogeny with minimum evolution test and default parameters. AtETR1 (AAA70047.1), AtERS1 (NP_181626.1), AtETR2 (NP_188956.1), AtERS2 (AAC62209.1), AtEIN4(AAD02485.1), AtCTR1 (AAA32780.1), AtEDR1 (AAG31143.1), AtRTE1(F4ITL6.1), AtRTE2(Q9SD42.1), AtEIN3 (NP_188713.1), AtEIL1 (NP_180273.1), AtEIL2 (NP_197611.1), AtEIL3 (NP_177514.1) and AtEIL4(NP_196574.1) in *Arabidopsis*; LeETR1 (AAC02213.1), LeETR2 (AAC02214.1), LeETR3 (AAC49124.1), LeETR4 (AAU34076.1), LeETR5 (AAD31397.1), LeETR6 (AAL86614.1), LeCTR1(AAL87456.1), TCTR2 (CAA06334.1), LeCTR3 (AAR89820.1), LeCTR4 (AAR89822.1), LeEIL1 (AAK58857.1), LeEIL2 (AAK58858.1), LeEIL3 (AAK58859.1) and LeEIL4 (BAC99307.1) in *Solanum lycopersicum*; PmRTE (XP_008220470.1) in *Prunus mume*; ObRTE (XP_006654719.1) in *Oryza brachyantha*; CsRTE (XP_006475936.1) in *Citrus sinensis*; StRTE (XP_006349393.1) in *Solanum tuberosum*; SiRTE (XP_004961370.1) in *Setaria italic*; CaRTE (XP_004498900.1) in *Cicer arietinum*; FaRTE (XP_004291036.1) in *Fragaria vesca subsp*. *Vesca*; CsRTE1 (XP_004157357.1) in *Cucumis sativus*; CsRTE2 (XP_004141314.1) in *Cucumis sativus*; DcRTE1 (ADW80941.1) in *Dianthus caryophyllus*.

His kinase (HisKA), GAF and receiver domains (REC) are the key domains of ETR-like proteins; all four of the ETR-like proteins detected in the present study possessed transmembrane domains: MnETR1 and MnERS each had three transmembrane domains, in contrast with the four domains of both MnETR2 and MnEIN4 (Fig. 1). Within the ETR-like family, MnETR1 and MnERS were clustered into the ETR1 subfamily, while MnETR2 and MnEIN4 belonged to the ETR2 subfamily in the phylogenetic tree (Fig. 2A).

The CTR1-like gene (*MnCTR1*) isolated from *M*. *notabilis*, has a genomic sequence length of 6569 bp and a 17 exon/16 intron structure. The coding sequence (CDS) of *MnCTR1* is 2592 bp, encoding 863 amino acids with a predicted molecular mass of 93.95 kDa (Table 4). The amino acid sequence from 589 to 843 contains the serine/threonine protein kinase domain (Fig. 1).

Within the EIN3-like family, the sequence similarity matrix analysis of MnEIL amino acids revealed that sequence identity ranged from 40% to 82% (Table 5). MnEIL1 and MnEIL2 were most similar to each other (82% amino acid identity) and were located near each other on scaffold3287. Phylogenetic analysis of the deduced amino acid sequences suggested that MnEIL1 and 2, MnEIL3, and MnEIL4 could be divided into three groups (Fig. 2D).

**Table 5:** Sequence similarity matrix of MnEIN3-like proteins.

Gene name	MnEIL1	MnEIL2	MnEIL3	MnEIL4
MnEIL1	100%			
MnEIL2	82%	100%		
MnEIL3	59%	59%	100%	
MnEIL4	40%	51%	50%	100%

The RTE-like gene (*MnRTE*) isolated from *M*. *notabilis* has a genomic sequence length of 1016 bp and a 2 exon/1 intron structure. The CDS is 693 bp, encoding 230 amino acids with a predicted molecular mass of 25.96 kDa and an isoelectric point (pI) of 6.64 (Table 4). MnRTE is most closely related with AtRTE2 in the phylogenetic tree (Fig. 2C). MnRTE has four transmembrane domains, two of which are located in its N-terminus and another two near its C-terminus (Fig. 1).

### Tissue specic expression

**Fig 3:**
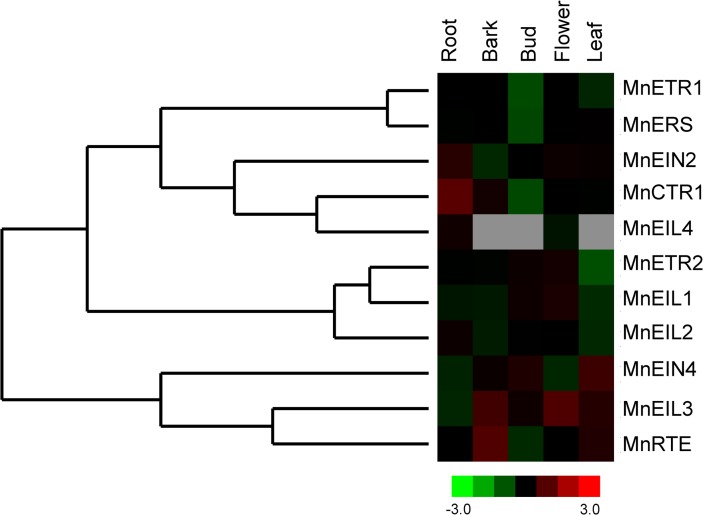
The expression profiles of genes involved in ethylene signal transduction in *Morus notabilis*. Expression analysis of genes involved in ethylene signal transduction based on the RPKM (reads per kilobase of exon model per million mapped reads) profile of five tissues (root, bark, bud, flower, and leaf). Cluster 3.0 software was used to normalize the expression level of the ethylene signal-related genes from RNA sequencing data. Sample names are shown above the heat maps. Color scale indicates the degree of expression: green, low expression; red, high expression; grey, no expression.

Reads per kilobase of exon model per million mapped reads (RPKM) was used to normalize the expression levels of the ethylene signal-related genes using *M*. *notabilis* RNA sequencing data. Expression of the 11 genes involved in ethylene signal transduction was detected in five tissues: root, bark, bud, flower, and leaf (Fig. 3).

Fig. 3 shows that the ethylene signal-related genes had similar expression patterns, except for *MnEIL4*, which had significantly lower expression levels in the root and flower (0.05 and 0.037 RPKM, respectively). *MnETR1*, *MnETR2*, *MnERS*, *MnEIN4*, *MnRTE*, *MnEIL1*, *MnEIL2*, and *MnEIL3* were expressed primarily in the bark and more weakly in the root, bud, flower, and leaf. However, *MnEIN2* and *MnCTR1* were more highly expressed in the root than in other tissues.

### Changes in fruit firmness, SSC and ethylene production during fruit development and harvest

**Fig 4:**
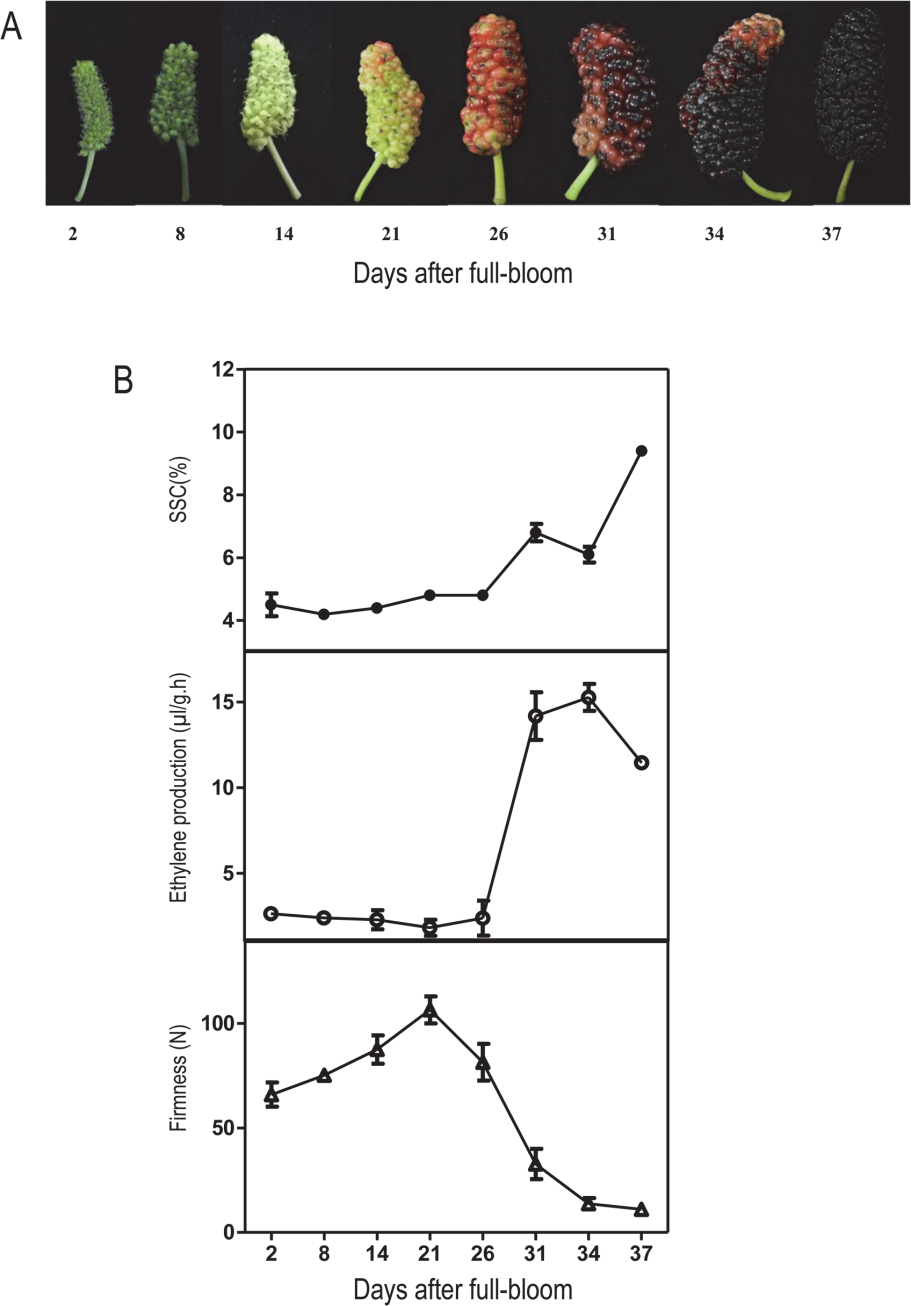
Changes in ethylene production, SSC and firmness during fruit development of *Morus atropurpurea* cv. *Jialing* No.40. (A) *Morus* fruit during development. (B) The ethylene production, SSC and firmness. The batch 1 fruits were used for these experiments. Error bars on each column indicate SDs from eight replicates.

The ethylene production, SSC, and firmness were detected over the development of *Morus* fruit (Fig. 4B). Ethylene production and SSC were relatively stable before 26 DAF and then increased rapidly to peak at 34 DAF, while fruit firmness declined rapidly after 26 DAF. Thus, 26 DAF may represent the transition stage of *Morus* fruit, and *Morus* fruit are probably climacteric.

**Fig 5:**
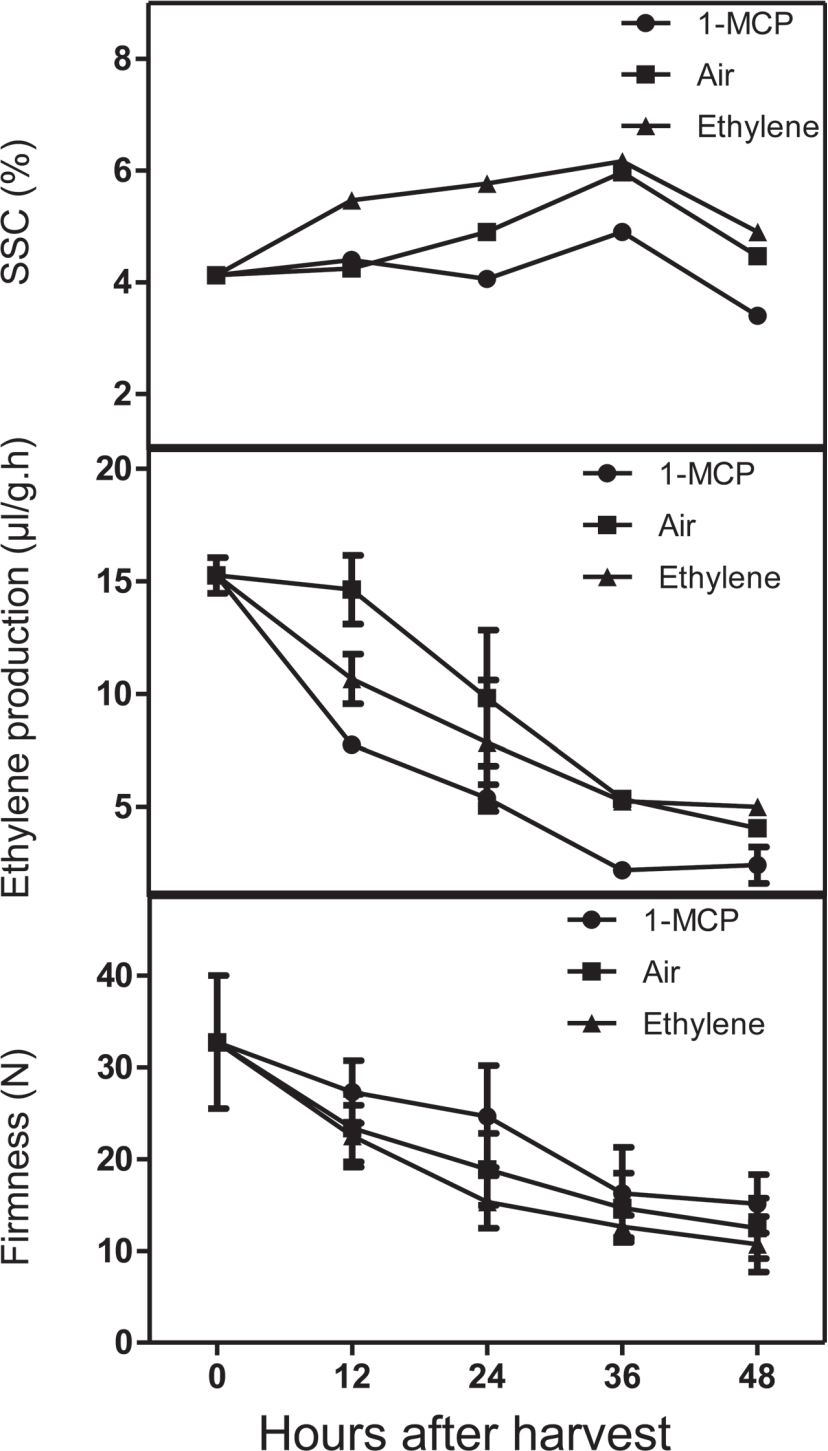
Effects of exogenous C_2_H_4_ and 1-MCP on postharvest fruit of *Morus atropurpurea* cv. *Jialing* No.40 at 23°C. The batch 2 fruits were divided into three groups. Two groups were treated with ethylene and 1-MCP, respectively. The third group of fruits was sealed in a similar container of the same volume as a control. Error bars on each column indicate SDs from eight replicates.

To investigate the response of fruit to exogenous ethylene and 1-MCP, the batch 2 fruits at 34 DAF were used. It is noteworthy to indicate that the days of growth required for fruit ripening varied between batches. The batch 2 fruit used here were at early ripening stage as revealed by SSC, ethylene production as well as fruit firmness (Fig. 5). Clearly, the production of ethylene decreased after harvest, and this decline was accelerated by 1-MCP (Fig. 5). Besides, slightly higher firmness and lower SSC were maintained in 1-MCP treated fruit (Fig. 5).

### Expression patterns during fruit development

**Fig 6:**
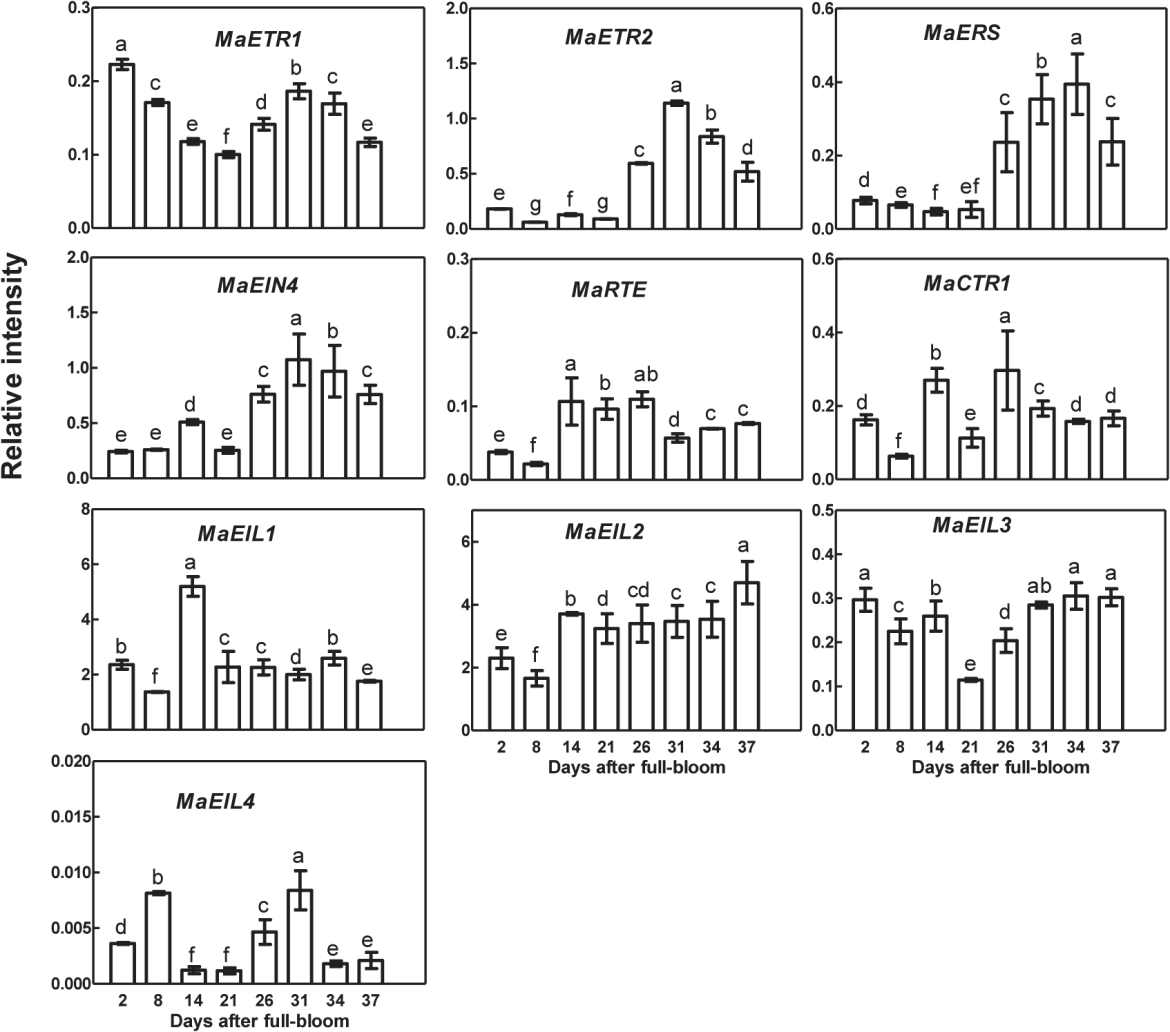
Expression of different components of the ethylene signaling pathway during fruit development of *Morus atropurpurea* cv. *Jialing* No.40. The batch 1 fruits were used for qRT-PCR. Each column height indicates relative mRNA abundance. Error bars on each column indicate SDs from three replicates. Significant differences (*P*<0.05) are marked with different letters above bars.

During development from 2 DAF through 37 DAF (to maturity), *MaETR*-like genes showed two expression patterns. *MaETR2*, *MaERS*, and *MaEIN4* had relatively low abundance in fruit at the early stages of development and higher abundance at 26 DAF (Fig. 6). Conversely, *MaETR1* was constantly expressed throughout fruit development, although it exhibited a strong expression peak at 2 DAF.

The expression patterns of *MaRTE* and *MaCTR1* were similar throughout fruit development, with relatively lower expression levels before 8 DAF and relatively higher levels at 14 DAF. The transcript levels from 31 DAF to 37 DAF were relatively constant and lower than those at 14 DAF and 26 DAF (Fig. 6).

Different patterns were observed among the *MaEIN3*-like family genes. *MaEIL1*, *MaEIL2*, and *MaEIL3* were constantly expressed during development, although the three genes exhibited a strong expression peak at 14 DAF, lower levels at 2 DAF and 8 DAF, and lower levels at 21 DAF, respectively. *MaEIL4* showed a different expression pattern, with much higher expression levels at 8 DAF and 31 DAF but lower levels at other stages (Fig. 6).

### Expression in response to ethylene and 1-MCP

**Fig 7:**
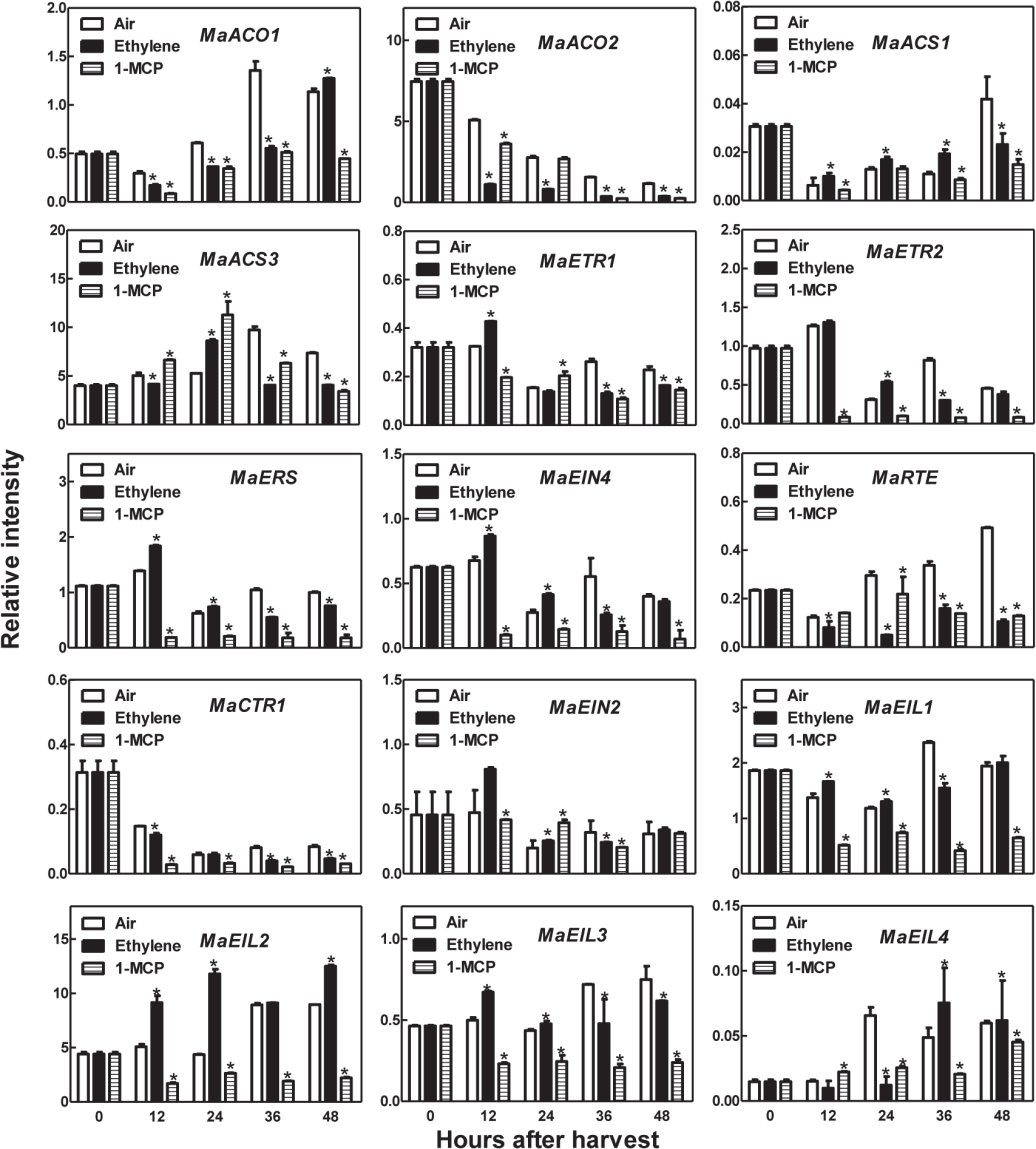
Expression of the genes involved in ethylene biosynthesis and signaling pathway during fruit ripening of *Morus atropurpurea* cv. *Jialing* No.40. The batch 2 fruits were used for qRT-PCR. Error bars on each column indicate SDs from three replicates. Significant differences (*P*<0.05) among treatment are marked with different letters above bars.

The transcript accumulations for two *MaACS* and two *MaACO* genes were investigated in the present study. During fruit storage, the transcript abundances of *MaACO1* and *MaACS3* increased while those of *MaACS1* and *MaACO2* decreased. The transcripts of *MaACO1*, *MaACO2*, and *MaACS3* were inhibited by ethylene, but those of *MaACS1* were upregulated during 12 to 36 hours after treatment. 1-MCP could downregulate the expression levels of *MaACO1*, *MaACO2*, *MaACS1* and *MaACS3* (Fig. 7).

The expression levels of all four *MaETR*-like genes increased transiently 12 h after harvest (HAH) and then decreased rapidly. The expression levels of *MaETR*-like genes were upregulated before 24 HAH and inhibited after 24 HAH by ethylene; expression was also significantly inhibited by 1-MCP during storage (Fig. 7).

The transcript abundances of *MaRTE* increased gradually during storage, but were inhibited by ethylene and 1-MCP. *MaCTR1* transcripts decreased during storage and were inhibited by ethylene and 1-MCP. *MaEIN2* was transiently upregulated by ethylene at 12 HAH but was not affected significantly by ethylene and 1-MCP (Fig. 7).

Among the *MaEIN3*-like genes, *MaEIL1* and *MaEIL3* were expressed relatively constantly, with little increase after harvest, while *MaEIL2* and *MaEIL4* expression increased rapidly after 24 HAH. All four genes were inhibited by 1-MCP and showed little response to ethylene, except for *MaEIL2* (Fig. 7).

## Discussion

*Morus* fruit is valued worldwide for its rich nutrient contents and unique flavor. However, the short maturity stages and shelf-life of *Morus* complicates efforts to popularize the fruit. The control of ethylene production may delay the ripening of *Morus* fruit and prolong its shelf life. However, no previous work has determined the respiratory type of *Morus* fruit, which is vital for establishing the mechanisms controlled by ethylene in *Morus* fruit development and ripening. In this study, ethylene production, firmness, and SSC were measured during the development of *Morus* fruit. These factors were relatively constant before 26 DAF but exhibited remarkable changes after this point. Ethylene production and SSC increased rapidly at 26 DAF, while fruit firmness decreased (Fig. 4). Our previous study suggested that *MaACO2* and *MaACS3* showed rapid increases in expression throughout fruit development and were upregulated by ethephon in the fruit at 20 DAF, which may be attributed to the production of ethylene during the fruit transitional period [27]. Therefore, fruit at 26 DAF may represent the transition stage from system I ethylene production to system II ethylene production, and the respiration rate during fruit development also supports this suggestion (S1 Fig.). In addition, ethylene production, fruit firmness, and SSC responded to ethylene and 1-MCP in the fruit harvested at 34 DAF. 1-MCP acted as a negative factor by retarding fruit softening, while ethylene accelerated fruit maturation (Fig. 5). However, unlike typical climacteric fruits, exogenous ethylene slightly decreased, rather than stimulated, endogenous ethylene production as well as temporarily inhibited expression of *MaACO1*, *MaACO2*, and *MaACS3* in *Morus* fruit after harvest. Why this occurred remains unclear and more studies are needed in the future.

*Morus* fruit at 34 DAF were harvested for treatment, and the production of ethylene decreased during storage in both treated and untreated fruit. *MaACO1* and *MaACS3* expression increased after harvest, while *MaACS1* and *MaACO2* decreased. The transcript levels of *MaACO1*, *MaACO2*, and *MaACS3* were inhibited by ethylene and 1-MCP, while that of *MaACS1* upregulated (Fig. 7). These results are similar to those reported in fig fruit (*Ficus carica* L.). In the fig fruit harvested at the post-climacteric stage, which had higher ethylene contents, ethylene production fell during storage and was inhibited by propylene; 1-MCP inhibited the accumulation of *Fc-ACS1*, *Fc-ACS3*, and *Fc-ACO1* transcripts, but induced the accumulation of *Fc-ACS2* transcripts. Propylene induced the accumulation of *Fc-ACS1*, *Fc-ACS3*, and *Fc-ACO1* transcripts, but inhibited *Fc-ACS2* expression in the propylene treated fruit [29]. Additionally, as both fig and *Morus* fruits are collective, their mechanisms of ethylene biosynthesis and fruit maturation may differ from those of other fruits.

In the present study, 11 putative genes were identified in the *M*. *notabilis* genome as associated with ethylene signal transduction using bioinformatics tools. These were four ETR1-like proteins, an RTE-like protein, a CTR1-like protein, an EIN2-like protein, and four EIN3-like proteins (Table 3; Table 4). Their expression patterns were studied during the development and ripening of *M*. *atropurpurea* cv. *Jialing* No.40 fruit.

Among the *ETR*-like genes, the expression levels of *MaETR2*, *MaERS*, and *MaEIN4* increased during development and were associated with the production of ethylene (Fig. 6), which was similar to the genes found in plum (*Pc-ETR1a*) [16], tomato (*NR*, *LeETR4*, *LeETR5* and *LeETR*6) [12], and strawberry (*FaETR2*) [30]. Notably, the expression of *MaETR1* differed from that of other *ETR*-like genes; the gene was constantly expressed during fruit development and showed higher expression at the early stage of fruit development, as was observed for similar genes in tomato (*LeETR1* and *LeETR2*) [12], strawberry (*FaETR1* and *FaERS1*) [30] and persimmon (*DkERS1*) [31]. *MaETR2*, *MaERS*, and *MaEIN4* may thus be associated with the production of ethylene during the maturation of *Morus* fruit, while *MaETR1* additionally contributes to the growth of immature fruit.

During fruit ripening, the expression levels of all four *MaETR*-like genes transiently increased at 12 HAH and then decreased rapidly, in conjunction with the trends of SSC and ethylene production after harvest (Figs. 5B and7). This result suggested that *MaETR* genes are closely related with the maturation of *Morus* fruit. The expression levels of *MaETR*-like genes were upregulated before 24 HAH and inhibited after 24 HAH in the ethylene treated fruit (Fig. 7). Ethylene thus likely accelerates the decrease of *MaETR* transcripts at post-climacteric stage.

RTE1 is a membrane protein located at the Golgi and endoplasmic reticulum (ER) that acts as a cofactor coordinated with ETR protein to negatively regulate the plant response to ethylene through a conformational effect on the ethylene-binding domain [32–34]. We are the first to report the expression pattern of the *RTE* gene during fruit development and maturation. *MaRTE* had relatively lower transcript abundance early in fruit development and higher abundance after 14 DAF (Fig. 6), which was similar to the expression patterns of *MaETRs*. We hypothesized that *MaRTE* regulates *Morus* fruit maturation in cooperation with *MaETR2*, *MaERS* and *MaEIN4*. *MaCTR1* expression was higher in ripe fruits, as has been reported in plum and pear [15–16], and showed a relatively similar pattern to that of *MaRTE*. The transcript abundance of *MaRTE*, unlike those of the *MaETR* genes, increased gradually after harvest, while that of *MaCTR1* decreased after harvest. However, *MaRTE* and *MaCTR1* expression levels were inhibited by ethylene and 1-MCP (Figs. 6 and 7).

The functions of *EIL* genes have been studied in tomato [22, 35], banana [36], melon [23], and kiwifruit [18, 24].Typically, *EIL* genes are constitutively expressed during fruit development, but *MA-EIL2* expression increases with the development of fruit and is induced by ethylene [36]. In our results, *MaEIL1*, *MaEIL2* and *MaEIL3* (but not *MaEIL4*) were constitutively expressed during fruit development, with slightly higher transcript abundances of *MaEIL1* and *MaEIL2* observed early in fruit development and a lower abundance of *MaEIL3* observed at 21 DAF. However, *MaEIL4* showed a different pattern with much higher expression levels early in development and at 31 DAF but lower levels at other stages (Fig. 6). In postharvest fruit, *MaEIL1* and *MaEIL3* were relatively constantly expressed, but were inhibited by 1-MCP, and showed little response to ethylene. Conversely, *MaEIL*2 and *MaEIL4* increased rapidly after 24 HAH and were upregulated by ethylene (Fig. 7). The functions of *MaEIL2* and *MaEIL4* during fruit ripening require further research.

In our previous study, the expression pattern of *MaEIN2* was determined and *MaEIN2* was showed to be involved in the ripening and senescence of *Morus* organs [37]. However, no significant change in the expression of *MaEIN2* was found during ripening or in response to ethylene and 1-MCP treatment in the present study (Fig. 7). The function of *MaEIN2* in fruit development and maturation requires further investigation.

In conclusion, we measured the ethylene production, fruit firmness and SSC during *Morus* fruit development and harvest, concluding that these fruit are probably climacteric. Analysis of *Morus* genome data characterized 11 elements associated with ethylene perception, and the genes involved in ethylene biosynthesis and signal transduction showed different expression patterns during *Morus* fruit development as well as different responses to ethylene and 1-MCP after harvest. The present study provides preliminary insights into the roles of ethylene and the expression profiles of ethylene-related genes during *Morus* fruit development, laying a foundation for a further understanding of the mechanisms of *Morus* fruit development and ripening. It may be possible to control *Morus* fruit ripening and artificially prolong its shelf life by manipulating ethylene production using transgenic approaches.

## Supporting Information

S1 Fig Changes in respiration rate during fruit development of *Morus atropurpurea* cv. *Jialing* No.40. The batch 3 fruits were used for measuring respiration rate. Error bars on each column indicate SDs from three replicates.
(DOCX)

S1 File Multiple sequence alignment of deduced *Morus notabilis* ethylene signaling proteins. The alignment was performed using CLUSTALX and the results were displayed using Genetyx 7.
(DOCX)

S2 File Multiple sequence alignment of ethylene signaling genes between *Morus notabilis* and *M*. *atropurpurea* cv. *Jialing* No.40. (DOCX)
