# Supplementary material for: Characterization and Expression of Genes Involved in the Ethylene Biosynthesis and Signal Transduction during Ripening of Mulberry Fruit
Source: PLoS One. 2015 Mar 30;10(3):e0122081. doi: 10.1371/journal.pone.0122081 (PMC4378970; doi:10.1371/journal.pone.0122081)
Supplement: S1 Fig — The batch 3 fruits were used for measuring respiration rate. Error bars on each column indicate SDs from three replicates. (DOCX) [file pone.0122081.s001.docx]

**S1 Fig. Changes in respiration rate during fruit development of *Morus atropurpurea* cv. *Jialing* No.40.** The batch 3 fruits were used for measuring respiration rate. Error bars on each column indicate SDs from three replicates.
