# Supplementary material for: Characterization and Expression of Genes Involved in the Ethylene Biosynthesis and Signal Transduction during Ripening of Mulberry Fruit
Source: PLoS One. 2015 Mar 30;10(3):e0122081. doi: 10.1371/journal.pone.0122081 (PMC4378970; doi:10.1371/journal.pone.0122081)
Supplement: S1 File — The alignment was performed using CLUSTALX and the results were displayed using Genetyx 7. (DOCX) [file pone.0122081.s002.docx]

**S1 Supporting Information. Multiple sequence alignment of deduced *Morus notabilis* ethylene signaling proteins.** The alignment was performed using CLUSTALX and the results were displayed using Genetyx 7.
