# Supplementary material for: Characterization and Expression of Genes Involved in the Ethylene Biosynthesis and Signal Transduction during Ripening of Mulberry Fruit
Source: PLoS One. 2015 Mar 30;10(3):e0122081. doi: 10.1371/journal.pone.0122081 (PMC4378970; doi:10.1371/journal.pone.0122081)

**S2 Supporting Information. Multiple sequence alignment of ethylene signaling genes between *Morus notabilis* and *M. atropurpurea* cv*. Jialing* No.40.**


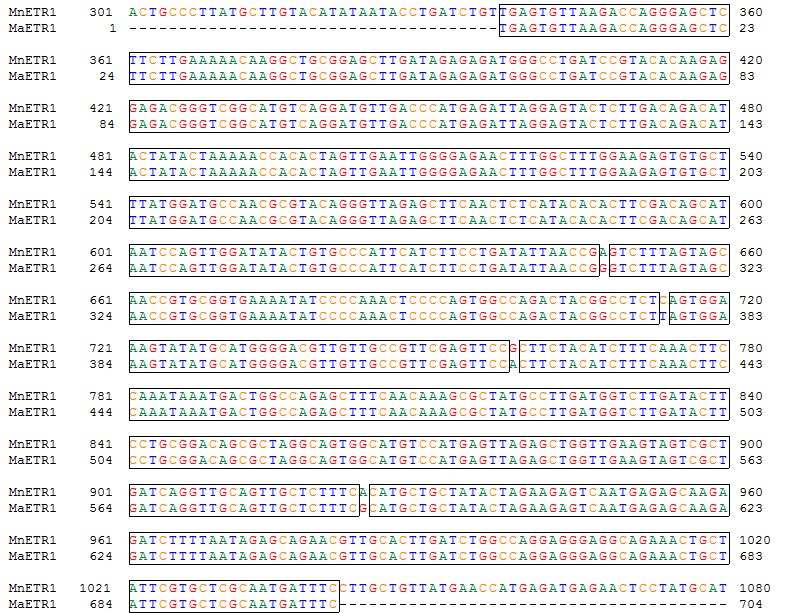


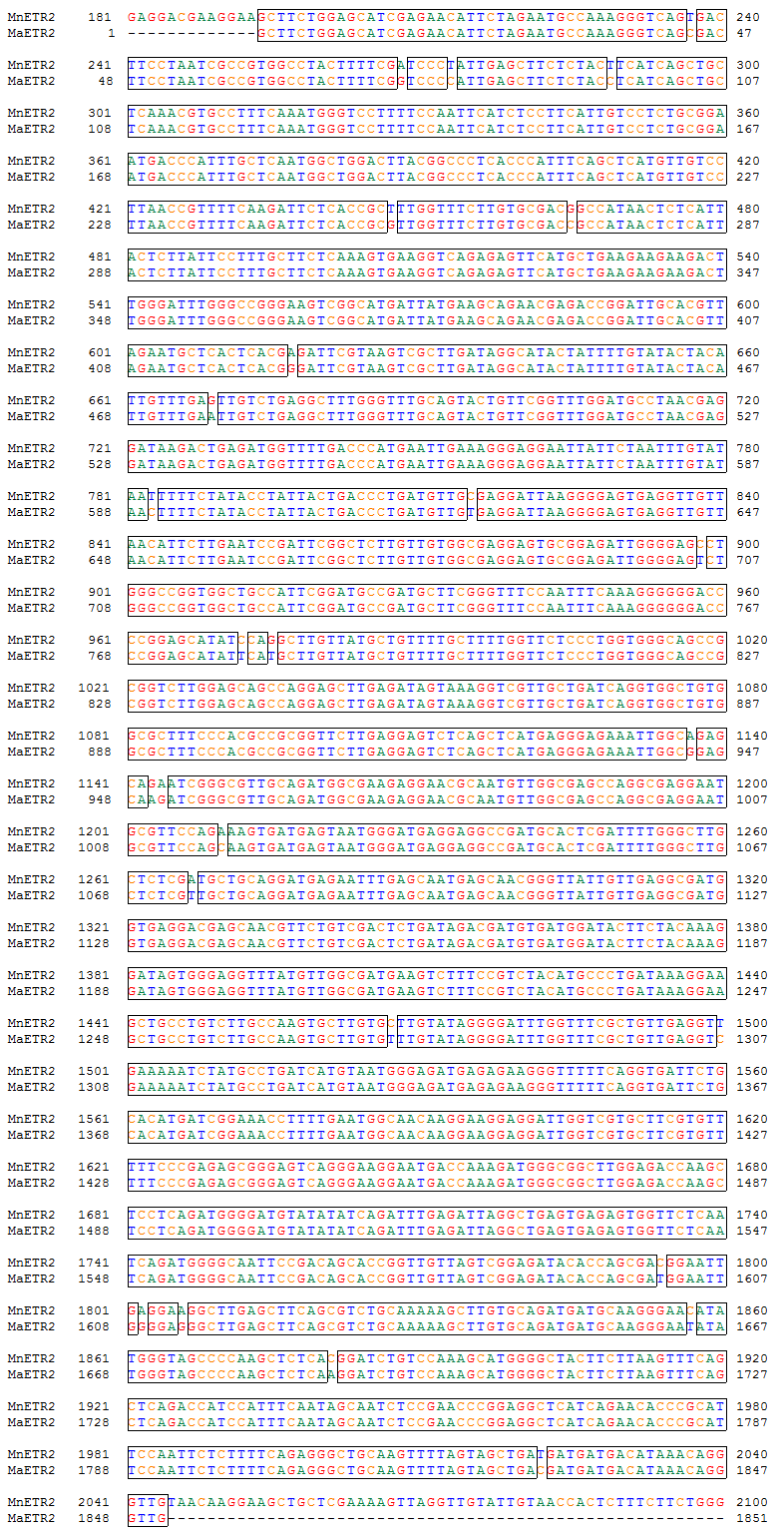


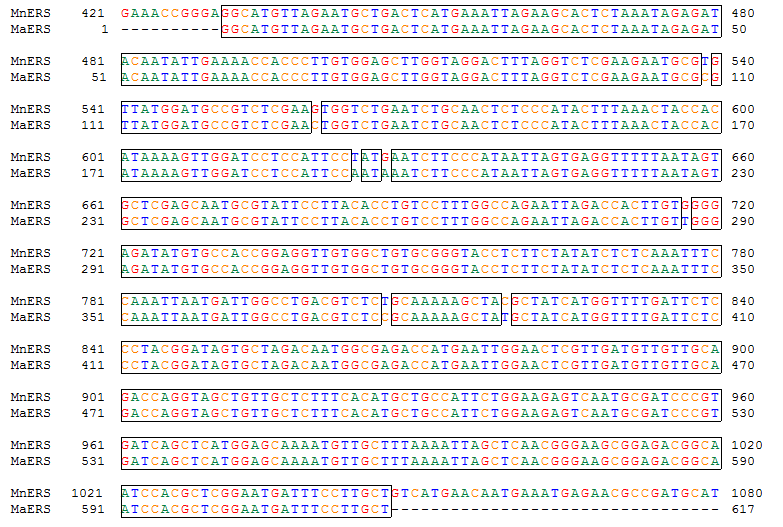


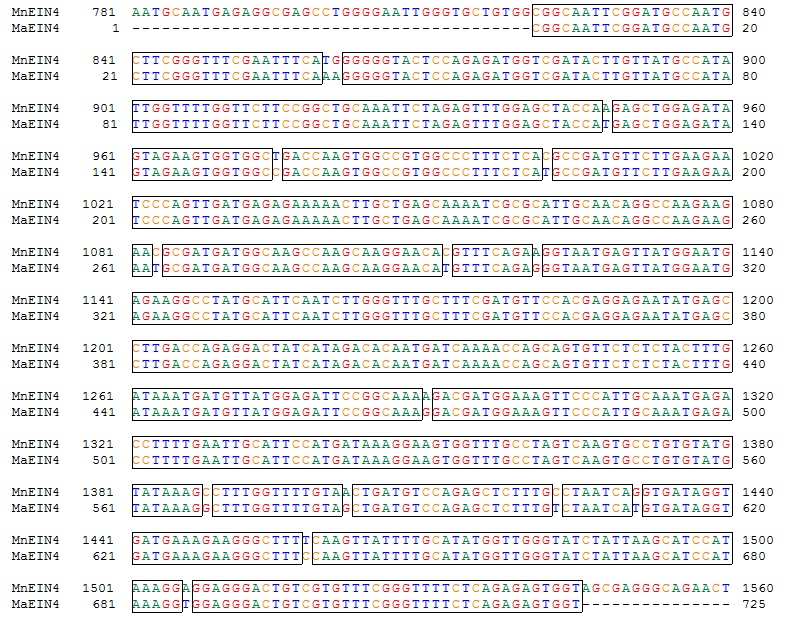

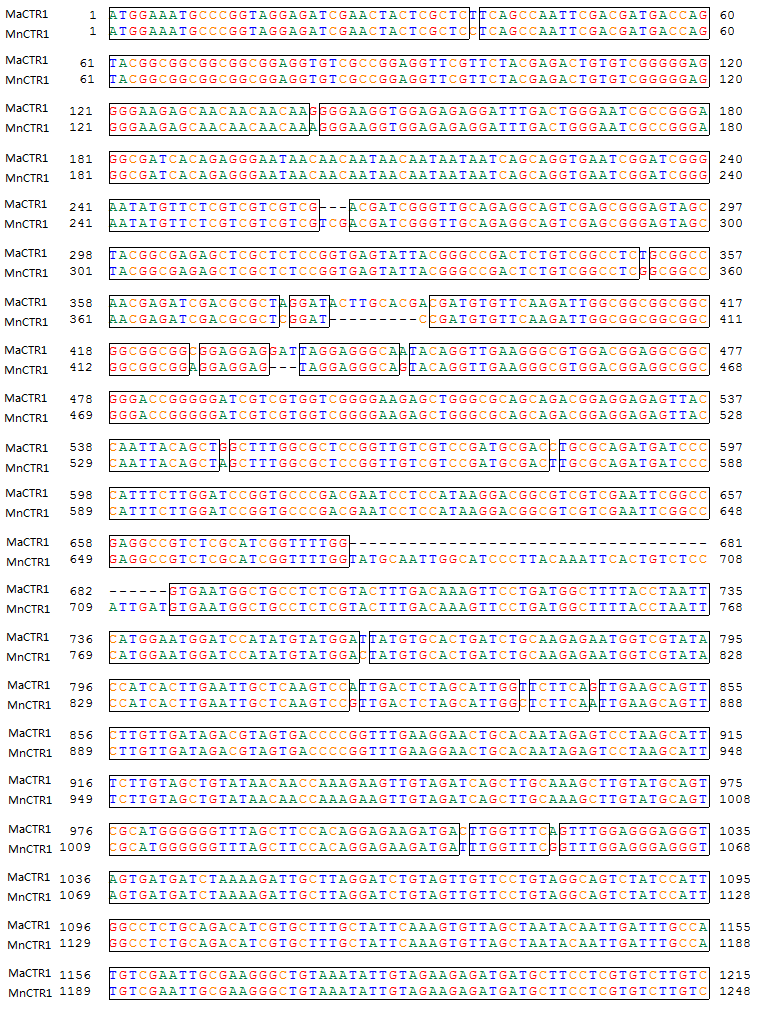


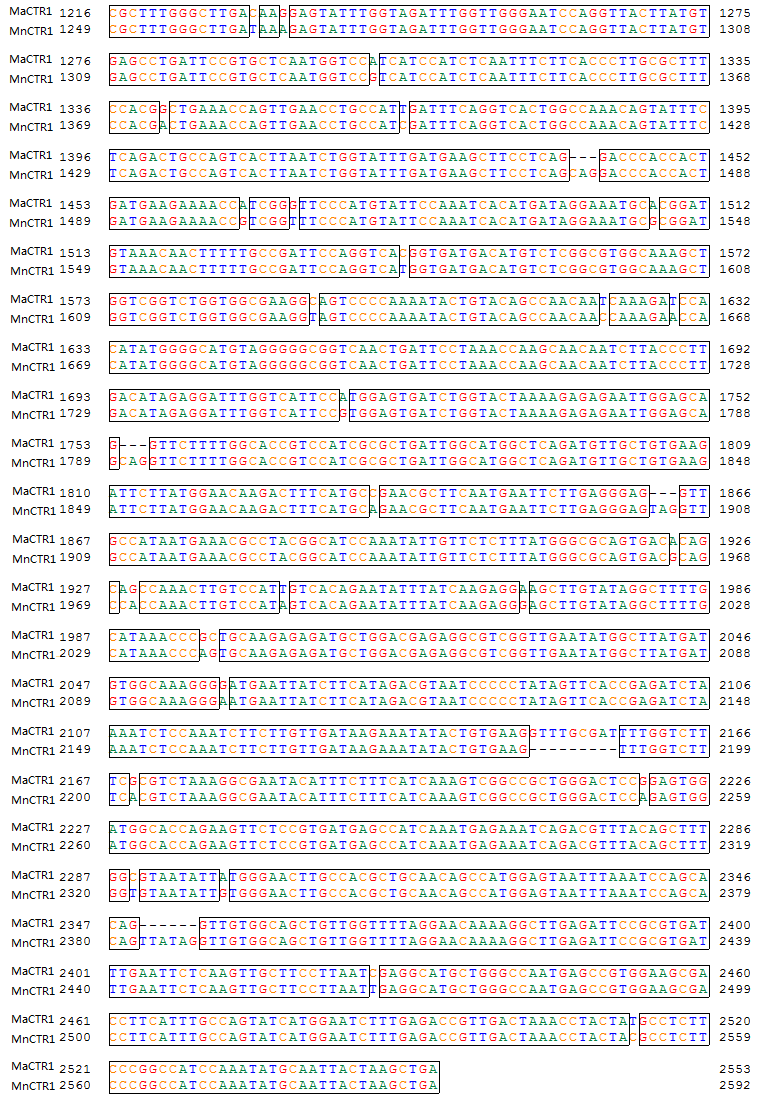

Supplement: S2 File — (DOCX) [file pone.0122081.s003.docx]
